# Invertase Suc2-mediated inulin catabolism is regulated at the transcript level in *Saccharomyces cerevisiae*

**DOI:** 10.1186/s12934-015-0243-3

**Published:** 2015-04-17

**Authors:** Fan Yang, Zhi-Cheng Liu, Xue Wang, Li-Li Li, Lan Yang, Wen-Zhu Tang, Zhi-Min Yu, Xianzhen Li

**Affiliations:** School of Biological Engineering, Dalian Polytechnic University, Dalian, 116034 PR China

**Keywords:** Invertase, Transcript level, *Saccharomyces cerevisiae*, Inulin

## Abstract

**Background:**

Invertase Suc2 was recently identified as a key hydrolase for inulin catabolism in *Saccharomyces cerevisiae*, whereas the Suc2 activity degrading inulin varies greatly in different *S. cerevisiae* strains. The molecular mechanism causing such variation remained obscure. The aim of this study is to investigate how Suc2 activity is regulated in *S. cerevisiae*.

**Results:**

The effect of *SUC2* expression level on inulin hydrolysis was investigated by introducing different *SUC2* genes or their corresponding promoters in *S. cerevisiae* strain BY4741 that can only weakly catabolize inulin. Both inulinase and invertase activities were increased with the rising *SUC2* expression level. Variation in the promoter sequence has an obvious effect on the transcript level of the *SUC2* gene. It was also found that the high expression level of *SUC2* was beneficial to inulin degradation and ethanol yield.

**Conclusions:**

Suc2-mediated inulin catabolism is regulated at transcript level in *S. cerevisiae*. Our work should be valuable for engineering advanced yeast strains in application of inulin for ethanol production.

**Electronic supplementary material:**

The online version of this article (doi:10.1186/s12934-015-0243-3) contains supplementary material, which is available to authorized users.

## Background

Inulin is a natural storage carbohydrate consisting of a linear β-2,1-linked D-fructofuranose chain terminated by a glucose residue through a sucrose-type linkage at the reducing end [1]. As an important material for biofuel such as ethanol production, inulin produced by Jerusalem artichoke has many advantages over other feedstock, e.g., cultivation of Jerusalem artichoke does not occupy farmland and Jerusalem artichoke has strong resistance to plant disease [2,3].

Although some yeast strains including *Kluyveromyces*, *Candida* and *Schizosaccharomyces* could directly ferment inulin without pretreatment, they are not so efficient for ethanol production [3-5]. *Saccharomyces cerevisiae* is the kind of efficient organism in regard of large-scale ethanol production, whereas most strains can not ferment large polymers of inulin [6]. Fortunately, several specific *S. cerevisiae* strains have been reported to have an ability to convert inulin-type sugars into ethanol without additional hydrolysis pretreatment [7-9]. Thus, here comes a question: what makes those inulin-degrading strains distinct from the others? Recently, the enzymatic hydrolysis of inulin in inulin-fermenting *S. cerevisiae* strain JZ1C was studied by Wang et al. [10]. An invertase Suc2, which was considered as a sucrose hydrolyzing enzyme, was purified and proved to be a key component able to degrade inulin for ethanol production in *S. cerevisiae* strain JZ1C [10]. It was also found that the amino acid sequence variation in Suc2 of strain JZ1C compared with that of weak-inulin-degrading strain S288c did not result in the change of specific enzyme activity [10]. Those results present the most possible explanation that the variation of *SUC2* transcription determines the inulin utilization traits of different *S. cerevisiae* strains.

*SUC2* transcript level in *S. cerevisiae* strains with different inulin-degrading activities was investigated in this study. Effect of transcription on the capability of catabolizing inulin in *S. cerevisiae* was determined by tuning the expression level of *SUC2* with different strength promoter. Our work aims to reveal the influence of the transcript level on Suc2 activity and the regulation of *SUC2* expression.

## Results

### Invertase Suc2 in different wild-type *S. cerevisiae* strains

**Figure 1:**
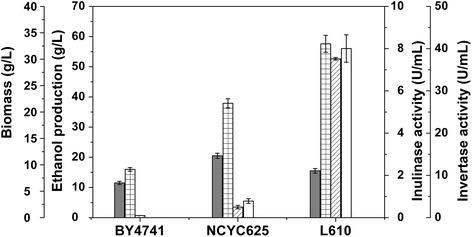
Inulin fermentation results of different wild-type yeast strains. Biomass (dry cell weight, gray), ethanol production (grid), inulinase activity (diagonal) and invertase activity (blank) were measured when *S. cerevisiae* strains BY4741, NCYC625 and L610 were cultured using inulin as a carbon source for 48 h. Error bars represent standard deviations from the mean of three biological samples.

As shown in Figure 1, the difference in ethanol production from inulin was observed in *S. cerevisiae* strain BY4741, NCYC625 and L610. Strain BY4741 produced low ethanol of 16 g/L, while strain NCYC625 produced higher ethanol of 38 g/L and strain L610 produced the highest ethanol of 58 g/L. The invertase and inulinase activities were well correlated with the ethanol productivity (Figure 1), suggesting that the high invertase activity was essential for ethanol production from inulin in *S. cerevisiae*. There was no considerable difference in the cell growth among those wild-type strains when incubated in the inulin medium. It was presumed that the great variation in invertase activity among those strains was attributed to the differences in *SUC2* genes or their corresponding regulation. Therefore, the cause for discrepancy in inulin utilization was investigated.

### Homologous *SUC2* ORFs encoding sucrose hydrolase had no difference in inulin-degrading activities

The previous report showed that the invertase Suc2 was responsible for the inulin degradation in *S. cerevisiae* [10], thus it was proposed that the Suc2 activities were largely varied in *S. cerevisiae* strains with different ability of inulin utilization. Such discrepancy in Suc2 activity may be attributed to the single nucleotide polymorphisms (SNPs) of encoding sequences [11] or the varied transcription level caused by difference of promoter strength [12].

**Figure 2:**
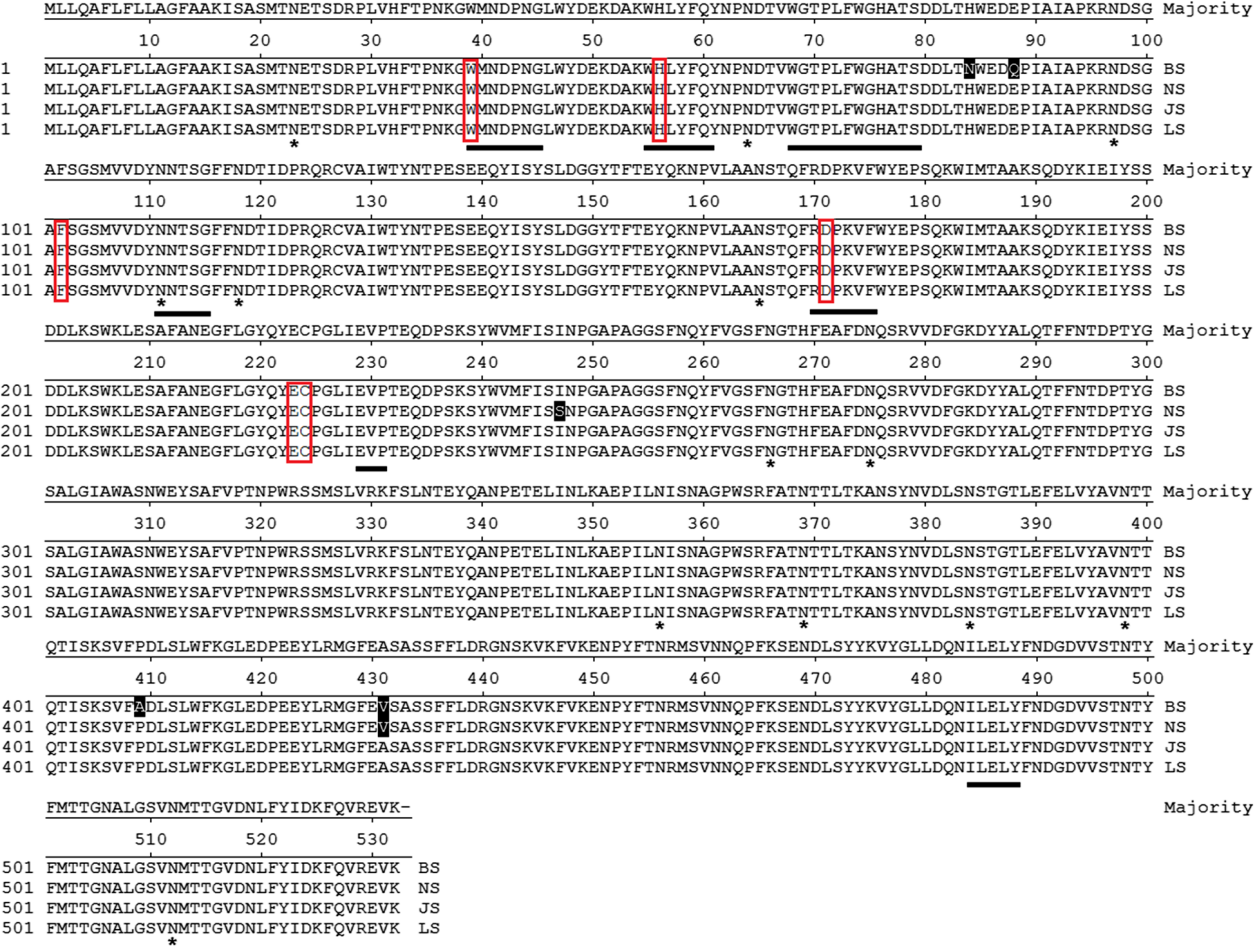
Amino acid sequence alignment of Suc2 from different *S. cerevisiae* strains. Amino acid variation in Suc2 from different *S. cerevisiae* strains BY4741 (BS), NCYC625 (NS), JZ1C (JS) and L610 (LS). Variable amino acid residues after sequence alignment were highlighted in black. “*” refers to N-glycosylation site. Substrate binding/catalysis sites were red boxed. High conserved regions were underlined.

The effect of SNPs on *SUC2* expression was evaluated as well as the performance of Suc2 in degrading inulin. The invertase encoding ORF *SUC2* was cloned from *S. cerevisiae* strains NCYC625 and L610. Their nucleotide sequences were compared with the reported sequences from *S. cerevisiae* strains BY4741 and strain JZ1C (Figure 2). Gene alignment showed that *SUC2* gene from strain L610 shared the same sequence with that from strain JZ1C (data not shown). Differences in amino acid sequence were observed in Suc2 from strains L610, NCYC625 and BY4741, whereas no sequence substitution in strains L610, NCYC625 and BY4741 was located at the substrate binding domain, catalytic domain or glycosylation site [13-15] (Figure 2).

**Figure 3:**
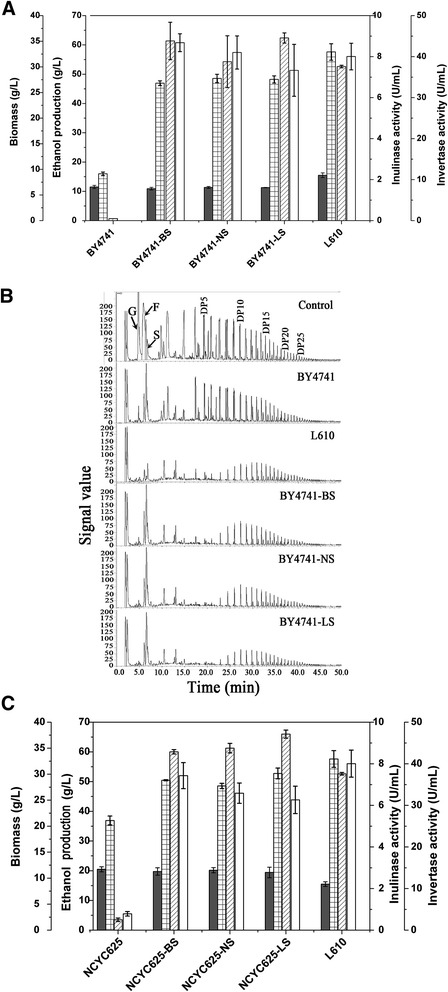
Inulin-fermenting performances of weak-inulin-utilizing yeast strains overexpressed with *SUC2.* **(A)** Biomass (dry cell weight, gray), ethanol production (grid), inulinase activity (diagonal) and invertase activity (blank) of strains BY4741 overexpressing different *SUC2* versions were assayed after cultured in inulin medium at 30°C for 48 h, in which BS, NS and LS indicated different *SUC2* versions from strains BY4741, NCYC625 and L610 respectively and error bars represent standard deviations from the mean of three biological samples; **(B)** Analysis of the inulin hydrolysate in the culture supernatant by ion chromatography, in which the supernatant of the 0-h culture was used as a control and all strains were cultivated in inulin medium for 48 h. Abbreviation G, F, and S represent glucose, fructose, and sucrose, respectively; **(C)** Biomass (dry cell weight, gray), ethanol production (grid), inulinase activity (diagonal) and invertase activity (blank) of strains NCYC625 overexpressing different *SUC2* versions were assayed, in which the culture condition, the indication of BS, NS and LS and the representation of error bars are the same as that of BY4741 recombinants.

In order to determine their performance for inulin hydrolysis, three different *SUC2* versions from strains L610 (LS), NCYC625 (NS) and BY4741 (BS) were expressed respectively in the weak-inulin-utilizing strains BY4741 and NCYC625. All the constructs were cultured in the inulin medium, and cell growth, ethanol production and enzyme activity were assayed. The activities of invertase and inulinase in BY4741 recombinants were comparable with that in strain L610 when different versions of *SUC2* gene were overexpressed in strain BY4741 under the control of constitutive *PGK1* promoter (Figure 3A). The increased ethanol production was also obtained in BY4741 recombinants, which was much higher than that in the control strain BY4741 (Figure 3A). However, the ethanol production by three recombinants was still lower than that by strain L610. The ion chromatography of the culture supernatant showed that recombinants BY4741-BS, BY4741-NS and BY4741-LS could efficiently ferment inulin with degree of polymerization less than 20, compared with the control strain BY4741 that can only assimilate glucose, fructose and sucrose (Figure 3B). The inulin-degrading activity and preference of BY4741 recombinants was comparable with that of the strain L610. Moreover, no obvious difference was observed in the inulin utilization profile when *SUC2* was overexpressed in recombinants BY4741 with different *SUC2* genes (Figure 3B).

Similarly, overexpression of different *SUC2* versions in strain NCYC625 also resulted in a dramatic increase in both enzyme activity and ethanol production from inulin (Figure 3C). Those results demonstrated that the constitutive overexpression of *SUC2* in the weak-inulin-utilizing strain endowed it with high ethanol production from inulin. The similar performance of three different *SUC2* versions suggested that the variation of sucrose hydrolase was not attributed to the SNPs of encoding sequences. Therefore, the transcription level of *SUC2* is a potential determinant of inulin utilization in *S. cerevisiae*.

### *SUC2* transcription level determines the activity of inulin degradation

**Figure 4:**
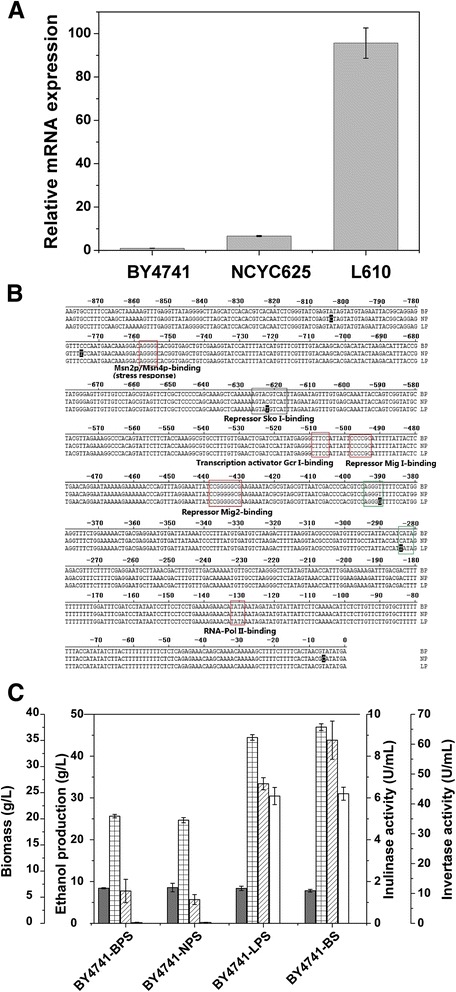
Effect of SNPs in the *SUC2* promoter on *SUC2* transcript level. **(A)** *SUC2* mRNA levels in *S. cerevisiae* strains BY4741, NCYC625 and L610 were determined by qRT-PCR, in which the *SUC2* transcription level in strain BY4741 was set as 1, while others in strains NCYC625 and L610 were compared with it and showed as relative data; **(B)** Sequence alignment of *SUC2* promoters from BY4741 (BP), NCYC625 (NP) and L610 (LP) by CLUSTAL W program. Important transcription factor binding sites were boxed in red. Nucleotide substitutions among three strains were blacked. A novel AGGGG sequence and a new TATA box formed by two nucleotide substitutions in *SUC2* promoter of stain L610 were boxed in green; **(C)** Biomass (dry cell weight, gray), ethanol production (grid), inulinase activity (diagonal) and invertase activity (blank) of strain BY4741 overexpressing *SUC2* under the control of different strength promoters were measured after cultured in inulin medium at 30°C for 48 h. Abbreviation BPS, NPS and LPS indicated *SUC2* expression under the control of the promoter from strain BY4741, NY4741 and L610 respectively, and BS indicated *SUC2* version from strain BY4741 under the control of the constitutive *PGK1* promoter. Error bars represent standard deviations from the mean of three biological samples.

In order to investigate the relationship between *SUC2* expression level and inulin utilization, the mRNA expression of *SUC2* was assayed by qRT-PCR in *S. cerevisiae* strains BY4741, NCYC625 and L610, which showed different inulin-degrading activity. As shown in Figure 4A, the *SUC2* transcript in inulin-utilizing strain L610 was much higher than that in weak-inulin-utilizing strains NCYC625 and BY4741. Such discrepancy of *SUC2* expression level in those three strains was in accordance with that of the invertase activity (Figure 1). Therefore, high expression of *SUC2* is essential for the inulin degradation in *S. cerevisiae*, and low expression of *SUC2* in weak-inulin-utilizing strain is presumably due to the promoter strength.

The nucleotide sequences of *SUC2* promoters from strains BY4741, NCYC625 and L610 were blasted using DNAStar MegAlign program. As shown in Figure 4B, there were three nucleotide substitutions in promoter sequence of strain L610 when compared with that of strains BY4741 and NCYC625.

Those three promoters were used to drive the expression of *SUC2* separately in strain BY4741. As shown in Figure 4C, *SUC2* expression under the control of the promoter from strain BY4741 (BPS) showed low ethanol production (26 g/L), inulinase (1.5 U/mL) and invertase activity (0.2 U/mL). Low ethanol production (25 g/L), inulinase (1.1 U/mL) and invertase activity (0.3 U/mL) were also obtained when *SUC2* expression was driven by the promoter from strain NCYC625 (NPS). However, the *SUC2* expression under the control of the promoter from strain L610 (LPS) showed high ethanol yield (44 g/L), inulinase activity (6.7 U/mL) and invertase activity (42.6 U/mL). Those results indicated that the promoter strength in driving the expression of *SUC2* determined the inulin-degrading capability of strains BY4741, NCYC625 and L610.

### Other factors have a minor effect on inulin utilization

**Figure 5:**
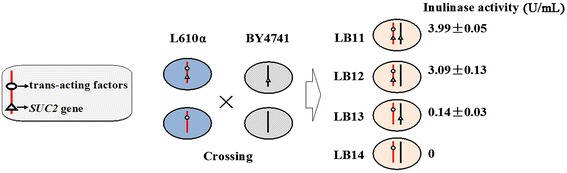
*SUC2/suc2* heterozygote construction and the inulinase activity. Mating experiment was performed between haploid strains L610α (blue) and BY4741 (gray) with or without *SUC2*. Four different heterozygotes, including L610α × BY4741 (LB11), L610α × BY4741 *suc2*Δ (LB12), L610α *suc2*Δ × BY4741 (LB13) and L610α *suc2*Δ × BY4741 *suc2*Δ (LB14), were produced. The inulinase activities were expressed as means ± standard deviation.

To elucidate whether *S. cerevisiae* strain L610 carries other genes or trans-acting factors responsible for inulin fermentation, haploid strain L610α and its mutant L610α *suc2*Δ were prepared and mated with strain BY4741 and mutant BY4741 *suc2*Δ, respectively. Four different heterozygotes were obtained, including L610α × BY4741 (LB11), L610α × BY4741 *suc2*Δ (LB12), L610α *suc2*Δ × BY4741 (LB13) and L610α *suc2*Δ × BY4741 *suc2*Δ (LB14). No inulinase activity could be detected in strains L610α *suc2*Δ and BY4741 *suc2*Δ (data not shown). As shown in Figure 5, the inulinase activity in heterozygote LB11 was comparable with that in LB12 whose *SUC2* allele from BY4741was deleted. Whereas trace-level inulinase activity was observed in strain LB13 when *SUC2* allele was deleted only from L610α, no enzyme activity remained in heterozygote LB14 when both *SUC2* alleles were deleted.

## Discussion

Invertase Suc2 (EC 3.2.1.26) belongs to sucrose-hydrolyzing enzyme but plays a key role in inulin catabolism by catalyzing inulin hydrolysis in yeast *S. cerevisiae* [10]. However, the inulinase activity of Suc2 can be detected only in some *S. cerevisiae* strains although *SUC2* is a constitutive genomic component [16]. As shown in Figure 1, all strains L610, NCYC625 and BY4741 could grow well in the inulin medium, whereas the Suc2 activity and ethanol production in strain L610 is much higher than that in strains NCYC625 and BY4741. It is presumed that biomass and ethanol production of strains NCYC625 and BY4741 is mainly from the residual glucose and fructose in the inulin medium but not from inulin (Figure 3B). In fact, a little inulin was catabolised by strain BY4741 compared with strain L610 after incubation in inulin medium for 48 h (Figure 3B). Therefore, both strains NCYC625 and BY4741 could weakly catabolize inulin and strain L610 could strongly utilize inulin for ethanol production.

It has been reported that *SUC2* gene encodes two different forms of invertase, external and internal enzyme. Internal invertase has no function in sucrose hydrolyzation, whereas the external invertase is excreted in the periplasmic space and plays the main role in sucrose hydrolysis [17,18]. It has been showed that the extracellular Suc2 is a glycosylated homodimer containing 14 potential N-linked glycosylation sites, in which eight sites are completely glycosylated and five sites are partially glycosylated [13,19]. Blast result of Suc2 sequences from *S. cerevisiae* strains BY4741, NCYC625, JZ1C and L610 showed five variations, including N84H, Q88E, A409P (BS vs. NS, JS, LS), V431A (BS, NS vs. JS, LS) and I247S (NS vs. BS, JS, LS). However, no variation in amino acid sequences was included in the glycosylation site (Figure 2). Therefore, the discrepancy in the inulinase activity should not be attributed to the glycosylation of invertase Suc2.

As previously reported [14,15,20-22], several conserved domains (underlined) and residues (Trp39, His56, Phe102, Asp171, Glu223 and Cys224) were predicted to be important for either substrate binding or catalysis (Figure 2). However, no variation in amino acid sequences in *S. cerevisiae* strains was located in those critical sites, indicating that the sequence changes in Suc2 were not the main reason for the discrepancy in enzyme activity.

It is significant that the overexpression of *SUC2* versions (LS, NS and BS) in strains BY4741 or NCYC625 makes the inulin-fermenting ability increase from zero to hero. The increased ethanol production from inulin with high degree of polymerization (around 48–53 g/L at 24 h) was obtained by all the *SUC2* overexpressing strains, which were higher than that by other inulin-fermenting wild *S. cerevisiae* strains, such as strain KCCM50549 (36.2 g/L at 34 h) [7]. The increased inulinase activity (around 8 U/mL) and invertase activity (31–43 U/mL) in recombinants was well correlated with the raised ethanol yield, which was much higher than that in strain JZ1C (around 1.5 U/mL for inulinase and 18 U/mL for invertase) [10]. All those results implied that the expression level of *SUC2* was probably the key factor for inulin catabolism in *S. cerevisiae*. Such speculation was confirmed by qRT-PCR results, which showed a progressive increase in *SUC2* transcript levels in order of strains BY4741, NCYC625 and L610 (Figure 4A).

The influence of promoter sequence variation on the expression level of *SUC2* was investigated. Several transcription factor binding sites have been reported to be located within the *SUC2* promoter, including TATA box, elements of catabolite repression (Mig1/Mig2 and Sko1 binding sequence) and transcriptional activation (Gcr1, Msn2p/Msn4p binding sites) [23-27]. As shown in Figure 4B, there are three nucleotide substitutions in the *SUC2* promoter of strain L610 when compared with that of strains BY4741 and NCYC625. The first nucleotide substitution in *SUC2* promoter of strain L610 was located in the position −627 to −617. Such mutation presumably resulted in the derepression of *SUC2* because the region between −627 and −617 acts as a negative element and can be recognized by repressor Sko1. The second nucleotide substitution in *SUC2* promoter of stain L610 formed a novel AGGGG (Msn2p/Msn4p binding) sequence in the position −390 to −394, which was called as STRE (stress response elements) and considered to be able to mediate transcription induced by environmental stress. The possible function of the third substitution in *SUC2* promoter of strain L610 might be related to the formation of a TATA box in position −281 to −284. As for the fermentation results, Suc2 in strain BY4741 under the control of the L610 *SUC2* promoter showed much higher enzyme activity than that under the control of other two promoters, which was in accordance with the presumption of promoter mutation. All those results suggested that the expression level of *SUC2* in *S. cerevisiae* strains was affected by the sequence variation in *SUC2* promoters, leading to different capability of inulin catabolism.

In order to confirm whether Suc2 is the only inulin-degrading enzyme in strain L610 and if there are any other elements responsible for inulin fermentation except the promoter, gene knock out and mating experiments were performed. No inulinase activity could be detected when *SUC2* gene in haploid strain L610α was deleted, suggesting that Suc2 is the key enzyme for inulin degradation in natural *S. cerevisiae* strains. When compared with strain BY4741 or heterozygote strain LB14, some inulinase activity was recovered in strain LB13, suggested that some trans-acting factors from L610α might have a weak interaction with cis-elements of *SUC2* allele from BY4741. Therefore, other elements had a minor effect on inulinase activity of Suc2 in addition to the *SUC2* promoter.

## Conclusions

The transcription level of *SUC2* is the key factor affecting the inulinase activity and inulin catabolism ability of *S. cerevisiae* strains. The sequence variation in *SUC2* promoter resulted in different transcript level of *SUC2* in different *S. cerevisiae* strains.

## Methods

### Strains and culture condition

*S. cerevisiae* BY4741 was a kind gift from Prof. Zhao (Dalian Institute of Chemical Physics, CAS). *S. cerevisiae* NCYC625 was purchased from China General Microbiological Culture Collection Centre. *S. cerevisiae* L610 was isolated as described previously [28]. Other information for S. cerevisiae strains used in this study was listed in Table 1. *Escherichia coli* DH5α was used in all cloning experiments.

**Table 1:** **Strains used in this study**

**Strain**	**Genotype**	**Source or reference**
BY4741	*MATa his3*Δ*1 leu2*Δ*0 met15*Δ*0 ura3*Δ*0*	A gift from Prof. Zhao Z
BY4741-BS	BY4741 containing pYC230-B-*SUC2* ^a^	This study
BY4741-NS	BY4741 containing pYC230-N-*SUC2* ^b^	This study
BY4741-LS	BY4741 containing pYC230-L-*SUC2* ^c^	This study
BY4741-BPS	BY4741 containing pYC230-BP-B*SUC2* ^d^	This study
BY4741-NPS	BY4741 containing pYC230-NP-B*SUC2*	This study
BY4741-LPS	BY4741 containing pYC230-LP-B*SUC2*	This study
NCYC625	Wild type, diploid	CGMCC
NCYC625-BS	NCYC625 containing pYC230-B*SUC2*	This study
NCYC625-NS	NCYC625 containing pYC230-N*SUC2*	This study
NCYC625-LS	NCYC625 containing pYC230-L*SUC2*	This study
L610	Wild type, diploid	Yang et al.
L610α	Haploid, MATα	This study
L610α *suc2*Δ	L610α in which *SUC2* gene was deleted	This study
BY4741 *suc2*Δ	BY4741 in which *SUC2* gene was deleted	This study
LB11	Heterozygote: L610α × BY4741	This study
LB12	Heterozygote: L610α × BY4741 *suc2*Δ	This study
LB13	Heterozygote: L610α *suc2*Δ × BY4741	This study
LB14	Heterozygote: L610α *suc2*Δ × BY4741 *suc2*Δ	This study

^a^B-*SUC2* indicates the *SUC2* gene of BY4741;

^b^N indicates the strain NCYC625;

^c^L indicates the strain L610;

^d^BP indicates *PGK1* promoter in the plasmid pYC230-B-*SUC2* was replaced by the promoter of BY4741 *SUC2*.

*S. cerevisiae* BY4741, NCYC625 and their recombinant strains were routinely cultivated at 30°C in YPD medium (20 g/L glucose, 10 g/L yeast extract and 20 g/L peptone, pH 6.0) supplemented with 200 mg/L G418 if necessary. *E. coli* DH5α was grown at 37°C in Luria–Bertani (LB) medium (10 g/L tryptone, 5.0 g/L yeast extract, and 10 g/L sodium chloride, pH 7.0) supplemented with 100 mg/L ampicillin if necessary.

### Cloning of *SUC2* genes and promoters

The full-length *SUC2* genes and their corresponding promoters were amplified from the genomic DNA of *S. cerevisiae* strains BY4741, NCYC625 and L610 respectively, using PrimeSTAR HS DNA Polymerase (TaKaRa Bio Inc., Dalian, China) and primers listed in Additional file 1: Table S1. The PCR products were ligated into plasmid pMD18-T (TaKaRa Bio Inc., Dalian, China) and sequenced.

Alignment of multiple nucleotide/amino acid sequences was performed using the CLUSTAL W program [29].

### Plasmid construction and yeast transformation

For overexpression assay in strains BY4741 and NCYC625, *SUC2* gene of strain BY4741 in the plasmid pYC230-B-*SUC2* (a kind gift from Fu-Li LI, Qingdao Institute of BioEnergy and Bioprocess Technology, CAS) was replaced by the *SUC2* gene of strain NCYC625 or strain L610 using a restriction-free (RF) cloning method described before [30]. To observe the effect of promoter on *SUC2* transcription, the *PGK1* promoter in the plasmid pYC230-B-*SUC2* was replaced by the *SUC2* promoters in strain BY4741, NCYC625 or L610, respectively, using RF cloning method. All primers used for plasmid construction were listed in Additional file 1: Table S1. All plasmids were confirmed by restriction enzyme digestion and sequencing. All constructs were transformed into *S. cerevisiae* strains according to the electroporation protocol [31].

### *SUC2* expression analysis

RNA was extracted from *S. cerevisiae* strain BY4741, NCYC625 and L610 respectively using RNAiso Plus according to manufacturer’s instruction (TaKaRa Bio Inc.). Totally 100 ng RNA was reverse transcribed using PrimeScript RT reagent Kit with gDNA Eraser (TaKaRa Bio Inc.). The reverse transcription product was diluted to one-tenth and used as the template for quantitative real-time PCR (qRT-PCR) with TaKaRa PCR Thermal Cycler Dice Real Time System (TaKaRa Code.TP800). To normalize the cycle threshold values, the relative transcript level for the housekeeping gene *ACT1* was determined. The condition used for qRT-PCR was as follows: 95°C for 30 s, 40 cycles of 95°C for 5 s, 60°C for 30 s [32]. The primers used for qRT-PCR were listed in Additional file 1: Table S1.

### *SUC2*/*suc2* heterozygote construction

The haploid *S. cerevisiae* L610 strains were prepared by sporulation and tetrad dissection as described before [33]. The expected 2:2 pattern of L610a (MATa) and L610α (MATα) was identified by colony PCR, and primer pairs MAT-F/MAT-a or MAT-F/MAT-α (listed in Additional file 1: Table S1) were used. The condition for colony PCR was as follows: 95°C for 10 min, 30 cycles of 95°C for 45 s, 52°C for 40 s, 72°C 45 s.

The *SUC2* gene in haploid L610α and BY4741 (MATa) strains was deleted using a PCR-mediated gene disruption method based on homologous recombination as reported by Wang *et al.* [10], resulting in L610α *suc2*Δ and BY4741 *suc2*Δ mutants.

Mating experiment was performed according to the classical protocol [33]. In brief, single colonies of L610a and BY4741 with or without *SUC2* gene were mixed and incubated in fresh YPD for 4 h. The mixed culture was diluted and plated on an YPD agar plate and cultivated at 30°C. The hybridized diploid strains were identified by colony PCR using the primers MAT-F/MAT-a/MAT-α together.

### Ethanol production

After incubated in 50 mL YPD medium supplemented with 100 mg/L G418 if necessary at 30°C and 150 rev/min for 24 h, the culture of *S. cerevisiae* strains was inoculated in 50 mL inulin medium with an inoculum of 5% (v/v) and cultivated at 30°C for 48 h for ethanol production. Inulin medium contained 150 g/L inulin, 5 g/L yeast extract and 0.5 g/L MgSO_4_ at pH 6.0. The culture was centrifuged at 4°C and 8000 g for 10 min and the supernatant was ready for measuring ethanol concentration and inulin degradation.

### Analysis of biomass and ethanol concentration

Biomass was determined by measuring the cell dry weight, which was obtained from cell pellet in 50 mL of culture broth, and dried in an oven at 105°C to a constant weight.

Ethanol concentration in the culture supernatant was measured by gas chromatography (Agilent Technologies Inc.) and both data acquisition and treatment were realized by the software Agilent ChemStation as described before [28].

### Enzyme assay

Inulinase or invertase activity was measured as described previously [10,34]. The reaction mixture consists of 50 μL cell culture and 450 μL of 2% inulin or sucrose in 100 mM acetate buffer (pH 5.0). The reaction was performed at 50°C for 15 min and terminated by boiling. The reducing sugar was assayed by the 3,5-dinitrosalicylic acid method [35]. One unit of enzyme activity was defined as the amount of enzyme that produced 1 μmol fructose per min under the assay condition used in this study.

### Ion chromatography

The degree of polymerization of inulin-type oligosaccharide in the culture supernatant before and after inulin fermentation by *S. cerevisiae* was analyzed by ion chromatography (Thermo Fisher Inc., ICS-5000) equipped with cation exchange analytical column Carbo Pac PA200 (250 mm × 3 mm) and cation exchange guard column Carbo Pac PA200 (50 mm × 3 mm). The column temperature was set at 30°C at a flow rate of 0.5 mL/min. The culture supernatant was diluted 100-fold and a volume of 25 μL of sample was injected. The oligosaccharides were separated by elution with 200 mM NaOH and 1 M NaOAc.

### Statistical analysis

All of the assays and determinations described in this paper were performed in triplicate unless otherwise stated. Data was expressed as the means of three biological samples ± standard deviation.

## Additional file

Additional file 1: Table S1. Primers used in this study. All the primers were synthesized by TaKaRa Bio Inc.
