# Supplementary material for: Invertase Suc2-mediated inulin catabolism is regulated at the transcript level in Saccharomyces cerevisiae
Source: Microb Cell Fact. 2015 Apr 17;14:59. doi: 10.1186/s12934-015-0243-3 (PMC4404613; doi:10.1186/s12934-015-0243-3)
Supplement: Additional file 1: Table S1. — Primers used in this study. All the primers were synthesized by TaKaRa Bio Inc. [file 12934_2015_243_MOESM1_ESM.docx]

Additional file 1: Table S1. Primers used in this study

| Name | Sequence (5’→3’) | Function |
| --- | --- | --- |
| SUC2-F | ATGCTTTTGCAAGCTTTCCTTTTC | *SUC2* amplification |
| SUC2-R | CTATTTTACTTCCCTTACTTGGAAC |  |
| SUC2-RF-F | CAAAAAGCTTTTCTTTTCACTAACGTATATGATGCTTTTGCAAGCTTTCCTTTTCC | Construction of pYC230-SUC2 |
| SUC2-RF-R | CGGATAAGAAAGCAACACCTGGCAATTCCTTAAGCTATTTTACTTCCCTTACTTGGAAC |  |
| P-SUC2-F | TTGAGTTAAGTGCCTTTCCAAGC； | *SUC2* promoter amplification |
| P-SUC2-R | AACCCACTCGTGTTGTTGTAATC |  |
| P-SUC2-RF-F | GTCGACACAATGACAGGTGTCATTTGAGTTAAGTGCCTTTCCAAGC | Construction of pYC230-P-SUC2 |
| P-SUC2-RF-R | CTATAGAGCCCTTAGGCAACATCATATACGTTAGTGAAAAGAAAAGC |  |
| ACT1-F | GCCGAAAGAATGCAAAAGGA | ACT1 RT-PCR |
| ACT1-R | GGAAGGTAGTCAAAGAAGCCAAGA |  |
| SUC2-F1 | TTTGGACCGTGGTAACTCTAAGG | SUC2 RT-PCR |
| SUC2-R1 | CAGACTTGAATGGTTGGTTGTTG |  |
| MAT-F | AGTCACATCAAGATCGTTTATGG | Mating type identification |
| MAT-α | GCACGGAATATGGGACTACTTCG |  |
| MAT-a | ACTCCACTTCAAGTAAGAGTTTG |  |
| SUCu-F | TACGCCCGATGTTTGCCTATTAC | *SUC2* gene disruption |
| SUCd-R | CATTACGATTGTGGGTTTATTACG |  |
| TKanT-RF-F | cgtatatgatgcttttgcaagcGACATGGAGGCCCAGAATAC |  |
| TKanT-RF-R | cctctattttacttcccttacttggCAGTATAGCGACCAGCATTCAC |  |
| Iden-u-f | CTAAGACATTTACCGTATGGGAG | *suc2Δ* mutants identification |
| Iden-u-r | TACGCTCGTCATCAAAATCACTCG |  |
| Iden-d-f | CGAGCGTAATGGCTGGCCTGTTG |  |
